# Comparison of Instantaneous Wave-Free Ratio (iFR) and Fractional Flow Reserve (FFR) with respect to Their Sensitivities to Cardiovascular Factors: A Computational Model-Based Study

**DOI:** 10.1155/2020/4094121

**Published:** 2020-05-11

**Authors:** Xinyang Ge, Youjun Liu, Zhaofang Yin, Shengxian Tu, Yuqi Fan, Yuri Vassilevski, Sergey Simakov, Fuyou Liang

**Affiliations:** ^1^School of Naval Architecture, Ocean and Civil Engineering, Shanghai Jiao Tong University, Shanghai 200240, China; ^2^College of Life Science and Bioengineering, Beijing University of Technology, Beijing 100124, China; ^3^Department of Cardiology, Shanghai Ninth People's Hospital, Shanghai Jiao Tong University School of Medicine, Shanghai 200011, China; ^4^Med-X Research Institute, School of Biomedical Engineering, Shanghai Jiao Tong University, Shanghai 200030, China; ^5^Institute for Personalized Medicine, Sechenov University, Moscow 119991, Russia; ^6^Moscow Institute of Physics and Technology, Dolgoprudny 141700, Russia; ^7^Institute of Numerical Mathematics, Russian Academy of Sciences, Moscow 119333, Russia

## Abstract

While coronary revascularization strategies guided by instantaneous wave-free ratio (iFR) are, in general, noninferior to those guided by fractional flow reserve (FFR) with respect to the rate of major adverse cardiac events at one-year follow-up in patients with stable angina or an acute coronary syndrome, the overall accuracy of diagnosis with iFR in large patient cohorts is about 80% compared with the diagnosis with FFR. So far, it remains incompletely understood what factors contribute to the discordant diagnosis between iFR and FFR. In this study, a computational method was used to systemically investigate the respective effects of various cardiovascular factors on FFR and iFR. The results showed that deterioration in aortic valve disease (e.g., regurgitation or stenosis) led to a marked decrease in iFR and a mild increase in FFR given fixed severity of coronary artery stenosis and that increasing coronary microvascular resistance caused a considerable increase in both iFR and FFR, but the degree of increase in iFR was lower than that in FFR. These findings suggest that there is a high probability of discordant diagnosis between iFR and FFR in patients with severe aortic valve disease or coronary microcirculation dysfunction.

## 1. Introduction

Fractional flow reserve (FFR), defined as the ratio between mean poststenosis coronary arterial and aortic blood pressures under a vasodilator-induced hyperemic condition [1], has been used as a gold standard for assessing the functional severity of epicardial coronary artery lesions in the past decades [2]. Recently, the instantaneous wave-free ratio (iFR), which can be measured without the need for vasodilator administration, has emerged as an alternative index of stenosis severity [3]. The concept of iFR is based on the hypothesis that there is a diastolic “wave-free” period (WFP) during the heartbeat period when coronary microvascular resistance is inherently stable and minimized [3]. In comparison with the measurement of FFR, measuring iFR is quicker and cheaper, and more importantly, it can avoid the potential side effects (e.g., breathlessness and chest tightness) associated with vasodilator infusion [3]. Clinical studies have shown that iFR is comparable to FFR with respect to diagnostic categorization [4] and that revascularization strategies guided by iFR are noninferior to those guided by FFR with respect to the risk of major adverse cardiac events at 12 months in patients with stable angina or acute coronary syndrome [5, 6]. A study comparing FFR and iFR with a third ischemic test (e.g., positron emission tomography myocardial perfusion imaging) as the arbiter showed that iFR did not perform differently from FFR in identifying hemodynamically significant ischemic coronary lesions [7]. On the other hand, some studies have revealed that iFR only correlated weakly with FFR in patients whose FFRs were in the clinically important range for decision making of 0.60 to 0.90 [8] and that the overall diagnostic accuracy of iFR (using a ROC-determined cutoff value of 0.90) was about 80% when FFR was used as the reference index for diagnosis (i.e., discordant diagnosis with iFR and FFR occurred in over 20% patients) [9]. So far, reasons underlying the discordant diagnosis between iFR and FFR remain incompletely elucidated. The study by Lee et al. [10] found that patients with discordant iFR and FFR (i.e., negative iFR while positive FFR) usually had higher hyperemic myocardial blood flow and CFR (coronary flow reserve) and higher resting microvascular resistance while there was greater reduction of coronary microvascular resistance at hyperemia compared to patients with concordant iFR and FFR, which implies that the resting state and hyperemic response of coronary microvasculature may be important factors related to the diagnostic agreement between iFR and FFR [11]. In addition, the functional status of the aortic valve has also been demonstrated to affect the diagnostic performance of iFR compared with FFR [12, 13]. For instance, it was found that the diagnostic accuracy of iFR in predicting an FFR of ≤0.8 was poor (65%) for coronary lesions in patients with severe aortic valve obstruction and tended to improve after transcatheter aortic valve implantation (TAVI) [13]. Despite the valuable insights from these clinical studies, a systemic analysis of the relationship between iFR and FFR under various pathophysiological conditions with identical severity of coronary artery disease remains absent due to the difficulties in completely removing the effects of interpatient variability and measuring all cardiovascular and hemodynamic parameters necessary for analysis in general clinical settings.

In comparison with in vivo measurements, computational modeling methods provide a more convenient approach to quantifying the impacts of any pathophysiological factors of interest on iFR and FFR at fixed severities of coronary artery disease and thereby establishing a basis for exploring mechanisms underlying the discordant diagnosis between iFR and FFR. Computational modeling methods have been widely applied in conjunction with medical image-based model reconstruction techniques to predict iFR and/or FFR [14–20]. While most model-based studies have demonstrated the ability of computational models to predict iFR and/or FFR with good accuracy in comparison with their in vivo counterparts, few studies have been dedicated to addressing the relationship between iFR and FFR over a wide range of pathophysiological conditions.

In the present study, a computational modeling method was employed to quantitatively investigate the respective sensitivities of iFR and FFR to various cardiovascular factors whose pathophysiological states are expected to differ among patients and have considerable influence on systemic and/or coronary hemodynamics and, based on this, identify the specific conditions under which iFR and FFR are most likely to give discordant diagnostic results.

## 2. Materials and Methods

### 2.1. Configuration of the Computational Model

**Figure 1:**
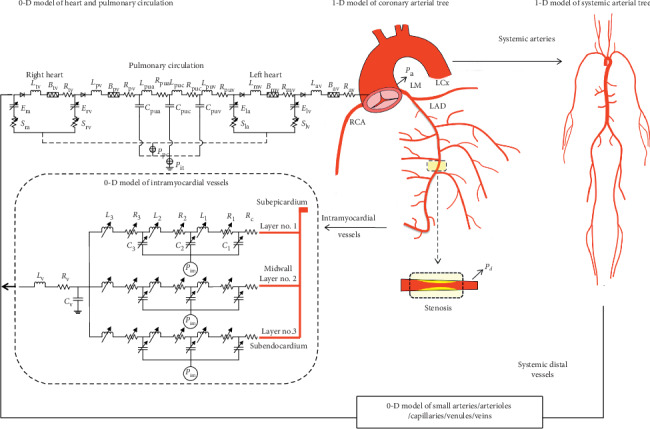
Schematic description of 0-1-D multiscale modeling of the coronary circulation coupled to the global cardiovascular system. Note that coronary branch arteries in the RCA and LCx territories were modeled but are not presented in the figure in order to save space. A stenosis was introduced in the middle segment of LAD, with blood pressure immediately distal to it (*P*_d_) being monitored along with blood pressure at the aortic root (*P*_a_) for the purpose of calculating iFR or FFR. More details of model development, parameter assignment, and numerical methods have been described in our previous studies [21, 22]. Abbreviations: LM, left main artery; LAD, left anterior descending coronary artery; LCx, left circumflex coronary artery; RCA, right coronary artery. Notations of main parameters: *L*, vascular inductance; *R*, vascular resistance; *C*, vascular compliance; *E*, elastance of cardiac chamber; *P*_it_, intrathoracic pressure; *P*_im_, intramyocardial tissue pressure.

The computational model was adapted from the models developed in our previous studies [21, 22] where the modeling methods and associated numerical schemes have been described in detail. In brief, a zero-one-dimensional (0-1-D) multiscale modeling method was employed to represent the coronary circulation coupled to the global cardiovascular system. 1-D modeling was applied to large epicardial coronary arteries and systemic arteries to describe pulse wave propagation and pressure/flow waveforms, while 0-D modeling was applied to intramyocardial vessels and the rest of the global cardiovascular system to describe intramyocardial and systemic hemodynamics. Coupling of the 1-D and 0-D models yielded a closed-loop model capable of describing both coronary and systemic hemodynamics as well as their interaction (see Figure 1). More importantly, the model provided a flexible platform for simulating iFR and FFR under various pathophysiological conditions through modifying the values of model parameters that represent various cardiovascular properties.

### 2.2. Parameter Assignment and Model Calibration

Model parameters were initially assigned based on population-averaged data reported in the literature [23, 24] to let model predictions fall in the ranges of in vivo hemodynamic data acquired from healthy subjects. It is noted that parameter assignment was implemented for nonhyperemic resting and hyperemic conditions, respectively, in order to meet the requirement for simulating iFR and FFR. Comparisons of model predictions and in vivo measurements under resting and hyperemic conditions are summarized in Table 1. In cases when aortic valve diseases (e.g., stenosis and regurgitation) were present, model parameters such as resting intramyocardial vascular resistance and systemic vascular resistance were further adjusted so that model-predicted coronary arterial flow and systemic arterial pressure were comparable to those measured in patients with aortic valve disease [25, 26] (refer to [21] for more details).

### 2.3. Definitions of iFR and FFR

iFR was defined as the ratio between the mean values of poststenosis coronary arterial and aortic blood pressures (herein denoted by *P*_d,wf_ and *P*_a,wf_, respectively) during the diastolic wave-free period (WFP) under the nonhyperemic resting condition [3]. Herein, WFP was set to begin in 25% of the way into diastole and end 5 ms before the end of diastole in accordance with the general definition of iFR in clinical practice [3]:

(1) $$\begin{array}{c} \text{iFR}=\frac{P_{\text{d,wf}}}{P_{\text{a,wf}}}. \end{array}$$

FFR was defined as the ratio between the mean poststenosis coronary arterial and aortic blood pressures (herein denoted by *P*_d,hp_ and *P*_a,hp_, respectively) during the entire cardiac cycle under the hyperemic condition [1]:

(2) $$\begin{array}{c} \text{FFR}=\frac{P_{\text{d,hp}}}{P_{\text{a,hp}}}. \end{array}$$

### 2.4. Baseline Computation Conditions

A stenosis was introduced in the middle segment of the left anterior descending coronary artery (LAD) (see Figure 1 for the location), with its length being fixed at 10 mm while the diameter stenosis rate (SR) varied from 0% (i.e., no stenosis) to 70% (i.e., severe stenosis). Heart rate (HR) was set to 66 beats/minute and 90 beats/minute for normal resting and hyperemic conditions, respectively.

### 2.5. Sensitivity Analyses of iFR and FFR with respect to Cardiovascular Factors

Physiologically, iFR and FFR could be affected by any cardiovascular factors involved in the regulation of coronary and/or systemic hemodynamics irrespective of whether they are related to the severity of coronary artery disease or not. In the present study, we considered six representative factors and categorized them into three groups: (1) cardiac factors, which include aortic valve function, the systolic and diastolic functions of the left ventricle, and heart rate (HR) that affects the magnitude and shape of aortic/cardiac blood pressure wave, as well as the extravascular tissue pressure of intramyocardial coronary vessels; (2) systemic vascular factors, which include the stiffness of the aorta and total systemic vascular resistance that affects the amplitude and mean value of aortic pressure wave, respectively; and (3) coronary vascular factors, which mainly include coronary microvascular resistance, a major determinant of trans-stenosis blood flow rate and pressure drop given coronary perfusion pressure and severity of stenosis.

#### 2.5.1. Parametric Representations of Cardiovascular Factors in the Model

All the aforementioned cardiovascular factors were represented in the model with parameters that can be quantitatively modified to reflect the variations in the pathophysiological states of the factors.

The status of aortic valve function was controlled by the effective orifice areas of the aortic valve during diastole and systole (herein denoted by EOA_dia_ and EOA_sys_, respectively). Assigning a value of >0 cm^2^ to EOA_dia_ represents the presence of aortic valve regurgitation (AR), whereas assigning a value lower than 4 cm^2^ (i.e., the normal value of EOA_sys_) to EOA_sys_ represents the presence of aortic valve stenosis (AS). Accordingly, progressively increasing EOA_dia_ (from 0 to 0.3 cm^2^) and reducing EOA_sys_ (from 4 to 1.0 cm^2^) represent the increasing severities of AR and AS, respectively. The systolic and diastolic functions of the left ventricle were parametrically represented by the peak systolic elastance (*E*_lva_) and baseline diastolic elastance (*E*_lvb_), respectively. Increasing *E*_lva_ represents the enhancement in myocardial contractility during systole, whereas increasing *E*_lvb_ represents the stiffening of the ventricular chamber (or impairment in myocardial relaxation) during diastole. HR was assigned directly in the model.

The stiffness of the aorta was controlled by the value assigned to the elastic modulus of the aortic wall in the model. Since the elastic modulus of the aortic wall is the main determinant of the aortic pulse wave velocity (aPWV), we herein took aPWV as a measure of aortic stiffness. An increase in aPWV corresponds to an increase in aortic stiffness. The total systemic vascular resistance (*R*_sys_) and coronary microvascular resistance (*R*_cmv_) are holistic descriptions of vascular resistances distributed in systemic tissues/organs and myocardium, respectively, and were modified by simultaneously varying all the corresponding vascular resistances.

#### 2.5.2. Quantification of the Sensitivities of iFR and FFR to Cardiovascular Factors

In order to investigate how iFR/FFR is affected by varying the pathophysiological state of each aforementioned cardiovascular factor, we incrementally changed the value (values) of the model parameter (parameters) corresponding to the factor while fixing other model parameters at their reference states. In other words, we performed a one-at-a-time parametric study using the computational model to evaluate the sensitivity of iFR/FFR with respect to each individual cardiovascular factor. The range of variations in each model parameter was estimated based on clinical data measured under the nonhyperemic resting condition [22, 27–40] and is listed along with its reference value in Table 2. It is noted that for the purpose of simplicity, we assumed that the ranges of parameter variations relative to their reference values under the hyperemic condition were the same as those assigned for the resting condition. In all the sensitivity analyses, the severity of the mid-LAD stenosis was fixed at 50% or 70%.

The percentage difference of computed iFR/FFR relative to its reference value (computed with all parameters being held at their reference states) was then calculated to evaluate the impact of varying each model parameter on iFR/FFR. It is noted that due to the differential physiological conditions under which iFR and FFR are measured, there were two sets of reference values of model parameters: (1) one set corresponding to the intact resting condition, and (2) the other set corresponding to the hyperemic condition.

## 3. Results

### 3.1. Changes in iFR and FFR with the Severity of Coronary Artery Stenosis and Typical Hemodynamic Characteristics during iFR Measurement

**Figure 2:**
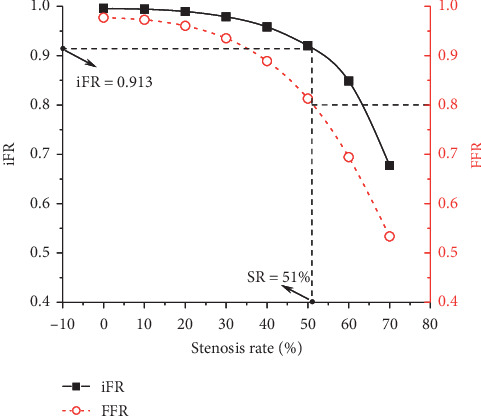
Model-simulated changes in iFR/FFR with the increase in the severity (i.e., the diameter stenosis rate is increased from 0% to 70% at an interval of 10%) of a stenosis present in mid-LAD under the control resting/hyperemic condition. When FFR is at the cutoff value (i.e., 0.8), the corresponding stenosis rate (SR) is 51% and iFR is 0.913.

Numerical simulations were firstly carried out to simulate iFR and FFR, respectively, with the severity of the mid-LAD stenosis being increased incrementally from 0% (no stenosis) to 70% (severe stenosis) while other cardiovascular factors fixed at their reference resting or hyperemic states. The simulated values of iFR and FFR both decreased monotonously with the severity of stenosis (see Figure 2). If a FFR of 0.8 was taken as the threshold for identifying a physiologically significant lesion [41], the corresponding iFR was 0.913, a value close to the cutoff value (0.89–0.93) established in previous clinical studies [12, 42, 43]. These results indicate that our model can reasonably predict the general relationship between iFR and FFR in the context of various severities of coronary artery stenosis.

**Figure 3:**
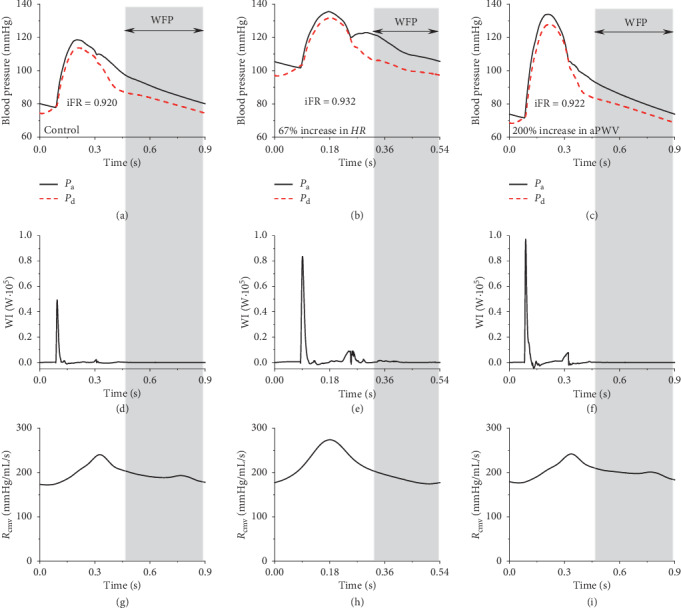
Model-simulated aortic and poststenosis coronary arterial pressure waves (a∼c), wave intensity in mid-LAD (d∼f), and poststenosis coronary microvascular resistance (g∼i) during iFR measurement under the control and two altered physiological conditions (one with a 67% increase in HR and the other with a 200% increase in aPWV). The wave-free period (WFP) during a cardiac cycle is highlighted by the gray shadow. The stenosis was present in mid-LAD, with the stenosis rate being fixed at 50% in all the simulations.

Figure 3 shows the model-simulated pressure waves in the ascending aorta and those immediately distal to a 50% stenosis in mid-LAD under the control condition (i.e., all model parameters were at the reference state) and under two altered physiological conditions characterized by a 67% increase in HR and a 200% elevation in aPWV, respectively. The wave-free pressure portions used to calculate iFR are highlighted by the gray shadows. Figure 3 also shows the corresponding time histories of wave intensity (WI) in the LAD (Figures 3(d)–3(f)) and total resistance of coronary vessels distal to the stenosis (Figures 3(g)–3(i)). As expected, the variations in HR and aPWV both led to considerable changes in pressure waveform and time history of WI via their influence on pressure wave propagation and reflection in the systemic arterial system, but they had little influence on iFR. In the wave-free period (WFP), WI was close to zero, proving that the “wave-free” assumption in the definition of iFR is reasonable; however, the poststenosis coronary vascular resistance was not constant during WFP. Nevertheless, the relatively low value of poststenosis vascular resistance during WFP compared with that in systole can still partly support the clinical hypothesis that iFR is an index derived under the condition of low coronary vascular resistance.

### 3.2. Sensitivities of iFR and FFR to Variations in the State of Each Cardiovascular Factor

**Figure 4:**
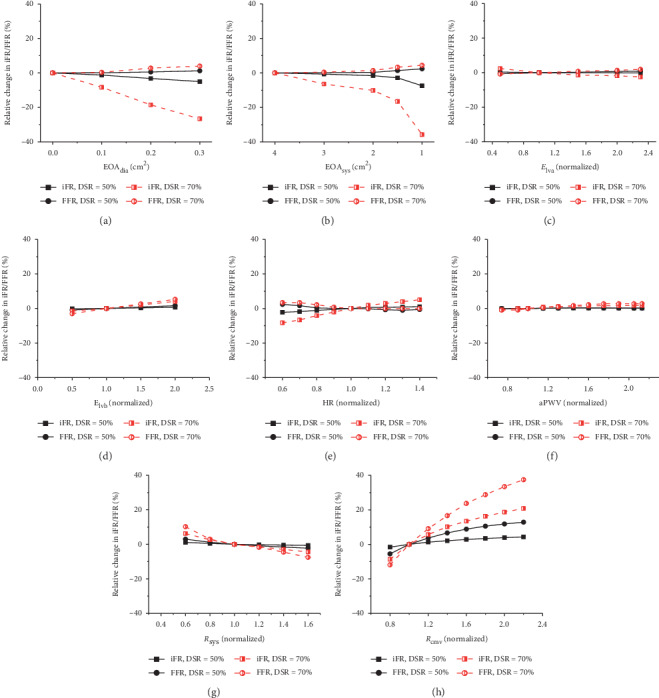
Percentage changes of iFR and FFR relative to their reference values upon the variations of each model parameter. The variations of all parameters except for EOA_dia_ and EOA_sys_ are expressed in normalized form relative to their reference values to facilitate the comparisons of the effects on iFR/FFR among different parameters. The stenosis is present in mid-LAD with its stenosis rate being set at 50% and 70%, respectively, and the corresponding reference values (computed with all model parameters being fixed at their reference states) of iFR/FFR are 0.920/0.813 and 0.677/0.534, respectively. (a) EOA_dia_. (b) EOA_sys_. (c) E_lva_. (d) E_lvb_. (e) HR. (f) aPWV. (g) R_sys_. (h) R_cmv_. Notations: EOA_dia_/EOA_sys_, effective orifice area of aortic valve during diastole/systole (an increase in EOA_dia_ represents an increase in the severity of aortic valve regurgitation, whereas a decrease in EOA_sys_ represents an increase in the severity of aortic valve stenosis); E_lva_/E_lvb_, peak systolic elastance/baseline diastolic elastance of the left ventricle; HR, heart rate; aPWV, aortic pulse wave velocity; R_sys_, total systemic vascular resistance; R_cmv_, total coronary microvascular resistance.

The sensitivities of iFR and FFR to variations in each of the eight model parameters that represent various cardiac or vascular factors are presented in the form of percentage changes relative to the reference values of iFR and FFR in Figure 4.

As for the sensitivities of iFR and FFR to cardiac factors (represented by EOA_dia_, EOA_sys_, *E*_lva_, *E*_lvb_, and HR in the model) (see Figures 4(a)–4(e)), iFR was observed to be highly sensitive to both EOA_dia_ and EOA_sys_ that represent the status of the aortic valve function, whereas FFR was only mildly affected by the variations in EOA_dia_ or EOA_sys_. Moreover, varying EOA_dia_ or EOA_sys_ induced opposite changes in iFR and FFR. For instance, increasing EOA_dia_ (representing a progressive deterioration in AR) or decreasing EOA_sys_ (representing a progressive deterioration in AS) remarkably reduced iFR whilst it elevated FFR mildly. When the results of the sensitivity analyses were further investigated with respect to the severity of coronary artery stenosis, an increase in stenosis rate (i.e., from 50% to 70%) was observed to considerably augment the sensitivities of iFR and FFR to EOA_dia_ and EOA_sys_. Relatively, both iFR and FFR were insensitive to the systolic and diastolic functions of the left ventricle (represented by *E*_lva_ and *E*_lvb_) and HR.

Varying the systemic vascular factors (i.e., aortic stiffness represented by aPWV and total systemic vascular resistance (*R*_sys_)) induced detectable while only mild changes in iFR and FFR (see Figures 4(f) and 4(g)). As is different from systemic vascular factors, increasing coronary microvascular resistance (*R*_cmv_) under the resting or hyperemic condition tended to significantly elevate iFR and FFR, although the degree of elevation in FFR was larger than that in iFR (see Figure 4(h)).

In summary, if a maximal percentage change in iFR or FFR of >10% in response to the variations in a model parameter was set as the threshold for judging high sensitivity, iFR was observed to be highly sensitive to EOA_dia_, EOA_sys_ (aortic valve function), and *R*_cmv_ (state of coronary microvasculature), whereas FFR was solely sensitive to *R*_cmv_.

### 3.3. Hemodynamic Characteristics Underlying the Differential Sensitivities of iFR and FFR to Aortic Valve Function and Coronary Microvascular Resistance

**Figure 5:**
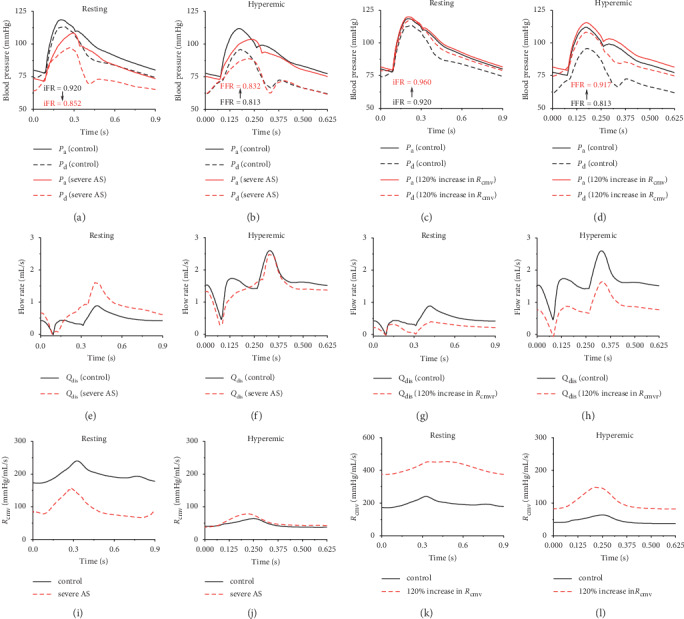
Comparisons of model-simulated aortic pressure wave and coronary arterial pressure wave distal to a 50% stenosis in mid-LAD and iFR/FFR (a∼d), flow wave in mid-LAD (e∼h), and poststenosis coronary microvascular resistance (i∼l) under control resting/hyperemic condition with those in the presence of severe AS (EOA_sys_ = 1.0 cm^2^) or increased coronary microvascular resistance (increased by 120% relative to the reference value).

In order to explore hemodynamic characteristics underlying the differential sensitivities of iFR and FFR to aortic valve function and coronary microvascular resistance, taking the 50% mid-LAD stenosis as an example, we plotted the model-simulated aortic pressure wave and poststenosis coronary arterial pressure/flow waves and poststenosis coronary microvascular resistance under the control condition (i.e., all model parameters were fixed at their reference states) against those under the condition characterized by the presence of severe aortic valve stenosis (AS) (represented by setting EOA_sys_ = 1.0 cm^2^) or increased coronary microvascular resistance (represented by increasing *R*_cmv_ by 120%) in Figure 5. It is noted that the numerical simulations were performed under the resting and hyperemic conditions, respectively, in consideration of the differential physiological conditions corresponding to iFR and FFR measurements.

Under the resting condition, although the presence of severe AS induced a marked decrease in both aortic and poststenosis coronary pressures, the degree of decrease in poststenosis pressure was larger than that of aortic pressure, resulting in an evident decrease in iFR. The enhanced decrease in poststenosis pressure was caused mainly by the increased resting coronary blood flow (which augments the pressure drop across the stenosis) as a consequence of coronary microvascular adaptive responses to increased myocardial stress and oxygen demand in the presence of AS [21]. Under the hyperemic condition, the simulated coronary blood flow rate in the presence of AS was however comparable to or even slightly lower than that under the control condition (which is consistent with previous clinical observations [44]), leading to a mild increase in FFR.

In contrast to AS, increasing coronary microvascular resistance under the resting or hyperemic condition had an overall small influence on the aortic pressure, but significantly elevated the poststenosis coronary pressure primarily due to its role in reducing trans-stenosis flow rate. Such effects were particularly pronounced under the hyperemic condition when the flow rate was higher and more sensitive to the variation in poststenosis coronary vascular resistance (see Figure 5(h)) compared with the resting condition, thereby leading to a larger increase in FFR than in iFR.

## 4. Discussion

In the present study, we employed a computational model to simulate the processes of iFR and FFR measurements and quantitatively investigated the respective sensitivities of iFR and FFR to various cardiovascular factors involved in the regulation of systemic and/or coronary hemodynamics. The results revealed that iFR and FFR differed considerably with respect to the cardiovascular factors to which they are sensitive and the degree and/or pattern of changes in response to the variations in the state of each cardiovascular factor.

The model-predicted marked decrease in iFR while mild increase in FFR following increasing severity of AS (simulated by reducing the value of EOA_sys_ in the model) implies that in patients with severe AS, the measured iFRs may be much lower than those in patients with equivalent severity of coronary artery disease while normal aortic valve function, although the measured FFRs in the two patient cohorts might be comparable, which may consequently lead to increased probability of discordant diagnosis between iFR and FFR in the former patient cohort if cutoff values of iFR and FFR established based on clinical data acquired from the latter patient cohort were used. These theoretical findings are consistent with relevant clinical observations reported in the literature. For instance, it was found that in patients with severe AS, the conventional iFR cutoff value had lower diagnostic agreement with FFR in the classification of coronary lesions and that a lower iFR cutoff value (e.g., shifting the cutoff value from 0.89 to 0.83) should be used in order to better predict a positive FFR [12, 13, 45, 46]. In the case of increasing severity of AR (simulated by increasing the value assigned to EOA_dia_ in the model), our study revealed similar patterns of differential changes in iFR and FFR to those found in the case of increasing severity of AS and would cause a similar trend of discordant diagnosis between iFR and FFR, although relevant clinical evidence from studies focused on patients with AR is rare, probably due to the low prevalence of AR in patients with coronary artery disease [47].

Unlike aortic valve disease which affects iFR and FFR in opposite ways, increasing coronary microvascular resistance led to a considerable increase in both iFR and FFR, although the degree of increase in iFR was lower than that in FFR. The differential effects of coronary microvascular resistance on iFR and FFR would become more evident when the resting coronary microvascular resistance is preserved while the hyperemic counterpart is higher than the normal value due to impaired vasodilation function, which may explain why low iFR and high FFR (i.e., iFR+/FFR−) were more frequently observed in patients with diabetes mellitus who usually have increased coronary microvascular resistance and low coronary flow at hyperemia due to microcirculation dysfunction [48, 49].

Relatively, varying left ventricular systolic and diastolic functions and HR and systemic vascular factors (i.e., aortic stiffness and systemic vascular resistance) over large ranges only had mild influences on iFR and FFR, which indicates that iFR and FFR would both perform well in assessing the functional severity of coronary artery lesions irrespective of potential high interpatient variability in these cardiac or vascular properties.

**Figure 6:**
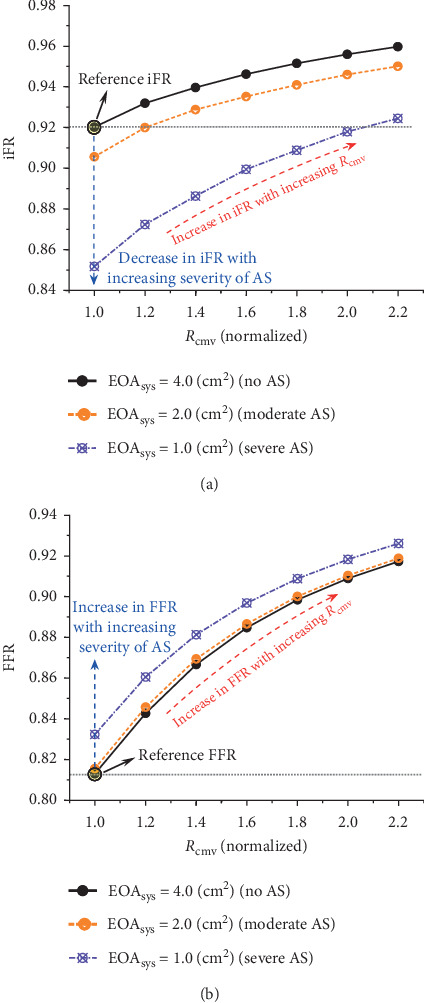
Effects of different combinations of AS (with its severity being controlled by the value of EOA_sys_) and increased coronary microvascular resistance (*R*_cmv_, herein normalized by its reference normal value) on (a) iFR and (b) FFR. Increasing the severity of AS leads to a marked decrease in iFR and moderate increase in FFR, whereas increasing *R*_cmv_ causes a progressive increase in both iFR and FFR. As a consequence, increasing *R*_cmv_ counteracts or even reverses the decrease in iFR while augments the increase in FFR caused by AS. Note that the coronary stenosis is present in mid-LAD with its severity being fixed at 50% in all the simulations and that the values of iFR and FFR highlighted by the filled circles indicate their reference values computed under the condition that only the 50% coronary stenosis is present while AS and increased *R*_cmv_ are absent.

In summary, the present study demonstrates the general trend that iFR and FFR are more likely to give discordant diagnostic results in the presence of severe aortic valve disease (stenosis or regurgitation) or increased coronary microvascular resistance. Therefore, special caution should be taken in the interpretation of measured iFR and FFR or the use of general cutoff values for diagnosis in patients with these specific cardiovascular conditions. Furthermore, given the differential effects on iFR and FFR of aortic valve disease and increased coronary microvascular resistance, the changes in iFR and FFR would become more complex in the presence of aortic valve disease combined with increased coronary microvascular resistance. Our additional numerical tests revealed that increasing coronary microvascular resistance could counteract or even reverse the decrease in iFR whilst augmenting the increase in FFR caused by aortic valve stenosis (see Figure 6). In this sense, in patients suffering from concomitant aortic valve disease and coronary microcirculation dysfunction, the diagnostic agreement between iFR and FFR could be highly complex and should be carefully interpreted in the context of patient-specific conditions.

## 5. Limitations

While our study, through quantifying the respective sensitivities of iFR and FFR to the variations in the pathophysiological state of each individual cardiovascular factor, provided useful insights for exploring mechanisms underlying the clinically observed discordant diagnosis between iFR and FFR in some patient cohorts, the study is limited by its theoretical nature and the focus on single-factor sensitivity analyses that render the findings unable to be applied directly to explain the measurements in individual patients whose cardiovascular conditions are highly complex and may deviate significantly from those represented by the model. In addition, the numerical simulations tailored to single-factor sensitivity analyses were not sufficient to generate a large database for statistical determination of the cutoff values of iFR or FFR under specific pathological conditions (e.g., various types and severities of aortic valve disease combined with other cardiovascular abnormalities). For this purpose, large-scale stochastic numerical simulations (similar to those reported in [50]) that cover a wide range of various pathophysiological conditions would be needed. The 0-1-D multiscale model employed in the present study is however computationally costly and therefore not well suited to such a study. The problem might be solved by developing a lumped-parameter model that contains the main components of the present model whilst is computationally much cheaper, which would be addressed in our future studies.

## 6. Conclusion

A computational model-based numerical study has been carried out to compare the sensitivities of iFR and FFR to variations in the pathophysiological states of various cardiovascular factors. It was found that aortic valve disease and increased coronary microvascular resistance had considerable while differential influences on iFR and FFR, which provides theoretical evidence for explaining the increased risk of discordant diagnosis between iFR and FFR in patients with aortic valve disease or coronary microcirculation dysfunction.

## Figures and Tables

**Table 1:** Comparisons of model simulations and in vivo measurements in terms of main systemic and coronary hemodynamic variables.

	Resting		Hyperemic	
	In vivo measurement	Simulation	In vivo measurement	Simulation
*Q*_LAD_ (mL/min)	76.15 ± 33.41 [24]	86.60	256.15 ± 110.84 [24]	264.91
*Q*_LCx_ (mL/min)	54.62 ± 24.59 [24]	64.40	163.85 ± 67.18 [24]	171.26
*Q*_RCA_ (mL/min)	68.46 ± 31.87 [24]	72.00	217.69 ± 76.70 [24]	232.54
*P*_as_ (mmHg)	113.0 ± 5.0 [23]	121.30	113.00 ± 6.0 [23]	111.68
*P*_ad_ (mmHg)	74.0 ± 8.0 [23]	79.70	70.00 ± 5.0 [23]	74.93
CO (L/min)	5.19 ± 0.83 [23]	5.14	7.6 ± 1.19 [23]	7.49

*Q*, mean flow rate over a cardiac cycle; *P*_as_/*P*_ad_, aortic systolic/diastolic pressure; CO, cardiac output.

**Table 2:** Reference values of model parameters involved in the sensitivity analyses for iFR and FFR under resting and hyperemic conditions.

Model parameter	Reference value (resting/hyperemic)	Range of variation (resting)
EOA_dia_ (cm^2^)	0.0/0.0	(0.0∼0.3) [27]
EOA_sys_ (cm^2^)	4.0/4.0	(4.0∼1.0) [28, 29]
*E*_lva_ (mmHg/ml)	2.87/2.87	(1.435∼6.601) [30]
*E*_lvb_ (mmHg/ml)	0.056/0.056	(0.028∼0.112) [30]
HR (beats/min)	66/90	(48∼111) [31, 32]
aPWV (m/s)	4.7/4.7	(3.478∼10.011) [33–35]
*R*_sys_ (mmHg·s/ml)	1.14/0.98	(0.456∼1.824) [36, 37]
*R*_cmv_ (mmHg·s/ml)	196.97/45.94	(157.58∼433.33) [38–40]

Note that the ranges of parameter variations under resting condition were estimated based on available clinical data reported in the literature.

## Data Availability

All clinical data used in the present study have been reported in the corresponding references and the values of most model parameters not provided in the manuscript have been reported in a previous study of ours [21] (https://onlinelibrary.wiley.com/doi/full/10.1002/cnm.3257).
